# Heterotopic Auxiliary Liver Transplantation With Portal Flow

**DOI:** 10.1155/1990/17682

**Published:** 1990

**Authors:** Laureano Lorente, Jaime Arias, Maria Angeles Aller, José Ignacio Ispizua, José Rodriguez, Hipólito Durán

**Affiliations:** 1 Departments of Surgery, Pathology and Surgical Research Hospital Universitario San Carlos USA; 2 Cátedra Patologia Quirúrgica Hospital Universitario San Carlos U.C.M., C/Martin Lagos s/n Madrid 28040 Spain

## Abstract

One of the causes of auxiliary liver transplantation failure is the inter-liver competition between the host liver and the graft for the hepatotrophic factors of the portal blood. We have developed an experimental model of heterotopic partial (30%) liver isotransplant using Wistar rats so as to study this competition.

Splenoportography and dissection demonstrate the existence of collateral circulation. The collaterals at 90 days post-transplant (PT) consisted of veins from the portal vein to the host liver (PR), paraesophageal veins (PE) and splenorenal veins (SR). At 60 days P.T., PR and SR veins but not PE ones appeared, and at 30 days P.T., there were only PR veins. Graft atrophy at 90 days P.T. was associated with a severe degree of bile duct proliferation.

The gradual development of portal hypertension causes porto-systemic collateral circulation and the graft loses the portal hepatotrophic factors. The late development of the portal hypertension and the biliary proliferation could be caused by the hepatic arterial ischemia in this experimental model. Thus, as has been described in the orthotopic liver tansplantation, the heterotopic one might require a double vascularization, both portal and arterial.


HPB Surgery, 1990, Vol. 2, pp. 281–293
Reprints available directly from the publisher
Photocopying permitted by license only
© 1990 Harwood Academic Publishers GmbH
Printed in the United Kingdom

### HETEROTOPIC AUXILIARY LIVER TRANSPLANTATION WITH PORTAL FLOW

### Gradual development of the Collateral Circulation

LAUREANO LORENTE, JAIME ARIAS, MARIA ANGELES ALLER, JOSÉ IGNACIO ISPIZUA, JOSÉ RODRIGUEZ, HIPÓLITO DURÁN
*Departments of Surgery, Pathology and Surgical Research, Hospital Universitario San Carlos.*

*(Received 29 November 1989)*

One of the causes of auxiliary liver transplantation failure is the inter-liver competition between the host liver and the graft for the hepatotrophic factors of the portal blood. We have developed an experimental model of heterotopic partial (30%) liver isotransplant using Wistar rats so as to study this competition.

Splenoportography and dissection demonstrate the existence of collateral circulation. The collaterals at 90 days post-transplant (PT) consisted of veins from the portal vein to the host liver (PR), paraesophageal veins (PE) and splenorenal veins (SR). At 60 days P.T., PR and SR veins but not PE ones appeared, and at 30 days P.T., there were only PR veins. Graft atrophy at 90 days P.T. was associated with a severe degree of bile duct proliferation.

The gradual development of portal hypertension causes porto-systemic collateral circulation and the graft loses the portal hepatotrophic factors. The late development of the portal hypertension and the biliary proliferation could be caused by the hepatic arterial ischemia in this experimental model. Thus, as has been described in the orthotopic liver tansplantation, the heterotopic one might require a double vascularization, both portal and arterial.

KEY WORDS: Heterotopic, liver transplantation biliary proliferation, porto-systemic collateral circulation.

### INTRODUCTION

Heterotopic or auxiliary liver transplantation (ALT) is an attractive alternative to the orthotopic liver transplant in patients with end-stage non-cancerous liver disease as well as for temporary support for a potentially reversible liver damage[1].

However, long-term survival after an ALT is exceptional[1–2]. One cause of ALT failure is the inter-liver competition of the host liver and the graft[3] for the use of various factors found in the portal blood[3–4]. The importance of the hepatotrophic factors, especially that of insulin, has been demonstrated by Starzl *et al.*[5] in dogs. Recently, reports in which the portal branch ligation model has been used have confirmed that hepatotrophic factors via the arterial supply prevent atrophy[7–8].

Address reprint requests to: Jaime Arias, M.D. 1 Cátedra Patologia Quirúrgica, Hospital Universitario San Carlos, U.C.M., C/Martin Lagos s/n, 28040 Madrid, España.

Based on the existence of hepatotrophic portal factors, many authors have proposed ALT techniques in rats, diverting the host portal blood into the transplant to avoid graft atrophy[4,9–12]. Based on this idea, we have developed an experimental model of heterotopic partial liver isotransplant in the rat so as to study the inter-liver competition.

### METHODS

Male Wistar rats weighing 250–350 g. were used as donors and recipients. The anaesthesia was maintained by ether inhalation. Atropine (0.1 mg) was injected i.m.

### *Donor Operation*

After entering the abdomen through a midline xiphopublic incision, the portal vein and bile duct were freed by dissection and the hepatic artery was ligated. A two-thirds hepatectomy was performed with the Higgins and Anderson method (1931)[13]. The vena cava was isolated and the right adrenal vein ligated and divided.

The liver was perfused through the portal vein with 15 ml of ice-cold Hartmann's solution to which 10,000 UI $L^{-1}$ of heparin had been added. During the perfusion, the suprahepatic vena cava was divided with a small cuff of diaphragm.

The infrahepatic vena cava was divided and finally the portal vein and the common bile duct were divided. The perfusion was stopped and the liver was immediately submerged into an ice-cold bath with Hartmann's solution. The donor operation lasted 12 to 16 minutes.

### *Cuff Preparation*

The cuffing procedure of the donor portal vein was done with Kamada's technique[14]. The cuff was made from a piece of polyethylene tubing (Vasocan 14G x 50 mm B. Braun Melsungen AG). The portal vein of the graft was everted over the cuff body and secured by ligature with 4–0 silk.

### *Recipient Operation*

A midline abdominal incision was made and the pyloric vein was ligated and divided to free the portal vein. Next the inferior vein cava (IVC) was dissected free between the renal veins. Clamping the IVC, a hole (0.4 x 0.3 cm) was cut the IVC, and the donor infrahepatic vein cava was anastomosed end-to-side with 8–0 silk suture. When the anastomosis was finished, the donor IVC was cross-clamped and the clamps of the recipient IVC were released. Then portal inflow to the graft was established by the end-to-end anastomosis cuff method (Figure 1). The bile duct was passed into the duodenum of the recipient and fixed to the wall by a simple stitch[15] (Figure 1). The recipient operation lasted 35 to 39 minutes.

No rat received antibiotic therapy. After a 6 hour fast, the animals were allowed access to food and water. The national and international laws were followed for the care and use of laboratory animals.

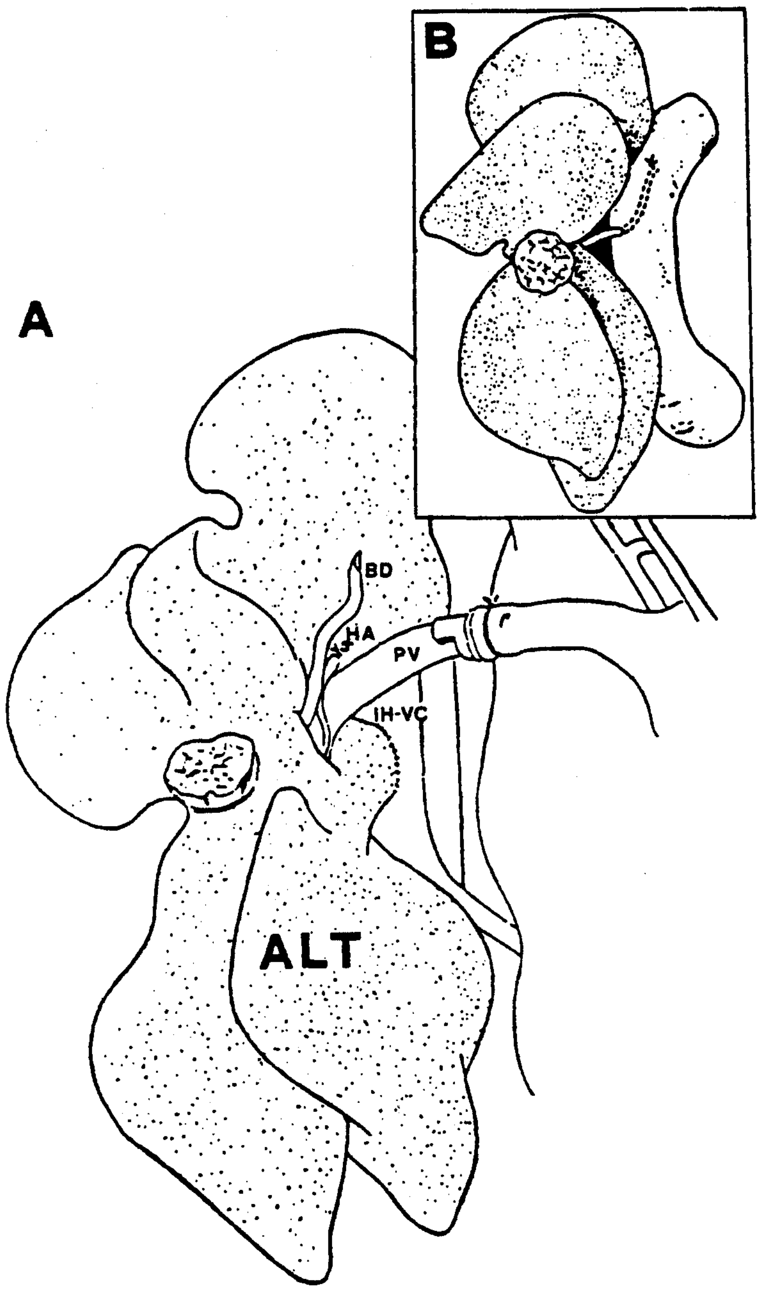

**Figure 1** Diagram of the auxiliary liver transplantation technique. A) ALT: auxiliary liver graft consist of right lateral and caudate lobes. Portal vein anastomosis (PV), infrahepatic vena cava anastomosis (IH–VC), bile duct (BD) and hepatic artery ligated (HA). B) Choledocho-duodenostomy.

### *Design of Experiments*

At 30 days (Group A; n = 8), 60 days (Group B; n = 8) and 90 days (Group C; n = 6) of the operation (ALT) the animals were studied in the following manner: after determinating the body weight and under ether anaesthesia, the abdomen was opened by a midline incision and a trans-splenic portal venography was performed by injecting 0.5 ml of Trazograf (R) Lab. Juste Spain in 2–3 seconds. A film was taken at the end of the injection.

Then, the areas of the porta hepatic, gastroesophageal junction, splenorenal and retroperitoneum were carefully observed for the presence of increased collateral veins and patency of the graft anastomosis. The animals were then exsanguinated by aortic puncture and the wet weights of the liver graft and the host liver taken. Both of these weights were compared with their respective weights preoperative as well as that of the liver in normal rats of similar body weights.

The graft and host liver were fixed in 10 per cent neutral formalin, dehydrated and embedded in paraffin. Liver sections were routinely treated with hematoxylin and eosin stain and silver stain.

The results were expressed as means ± SDs. The t-Test for unpaired samples were used for statistical analysis. P values less than 0.05 were considered significant.

### RESULTS

### *Splenoportography*

The portograms revealed the existence of collateral circulation in all the groups (A, B and C). These collaterals in group C occurred in the three basic areas described by Canty *et al.*[16]: a) veins from the portal vein to the host liver, producing portal revascularization (PR); b) paraesophageal veins (PE) and c) collaterals between the splenic vein and the left renal and adrenal system or spontaneous splenorenal (SR) shunts with retrograde filling of the inferior vein cava (Figure 2).

In group B, PR and SR veins but not PE appeared (Figure 2). Finally in group A, there were only PR veins (Figure 2).

### *Gross Anatomic Study*

Dissection of the majority of the isografts anastomosis was difficult because of the existence of adhesions. The permeability of all anastomosis was evident in groups

**Table 1** Development of the different collaterals circulation after auxiliary liver transplant in the rat.

| | *Portal* | *Spleno-renal* | *Paraesophageal* |
|---|---|---|---|
| 30 Days | *** | --- | --- |
| 60 Days | *** | *** | --- |
| 90 Days | *** | *** | *** |

(*** = yes) (--- = no).

A and B. We couldn't identify any lesions or obstruction in the choledocho-duodenostomy. Thrombotic occlusion of the portal vein was always demonstrated, in group C. Moreover, in this group, it was possible to identify the donor infrahepatic vein cava, in only three cases were there no thrombi at the anastomotic level.

In group A three animals presented fine collateral veins around the bile duct for the portal vein to the host liver hilium (PR). This type of collateral circulation was observed in six cases in group B, and in all the cases of group C.

In five of the cases in group B and all of the cases in group C, large and tortuous veins from the short gastric vessels to the left adrenal and renal veins (SR) were found. By direct visual examination, paraesophageal collaterals veins (PE) were only found in group C (Figure 3). A slight enlargement of the spleen was observed in four cases in group C.

### *Weights of Auxiliary and Host Liver*

The graft weight increased 140.2 ± 42.1% in group A and 15.2 ± 6.6% in group B in reference to their weight at operation. In group C, the isografts weights decreased 65.8 ± 1.8% (Figure 4).

All the host livers weighed less than at the preoperative level at 30, 60 and 90 days after the transplant (Figure 4) as well as the liver in normal rats of similar body weights.

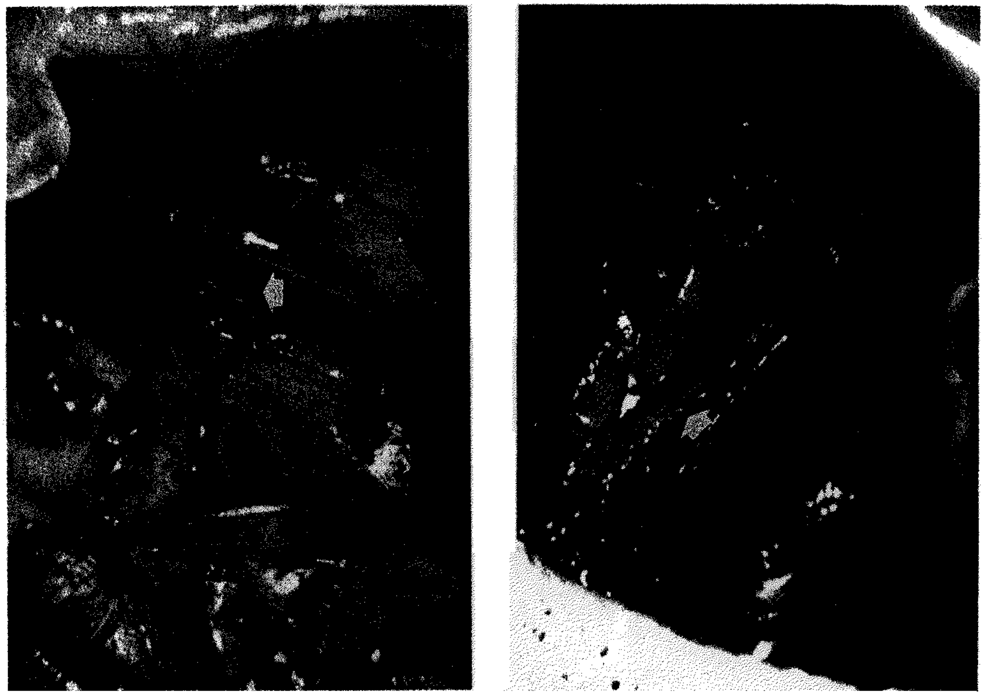

A B

**Figure 3** Gross anatomic aspect of the collaterals between the splenic vein and the left renal vein (A) and paraesophageal veins (B).

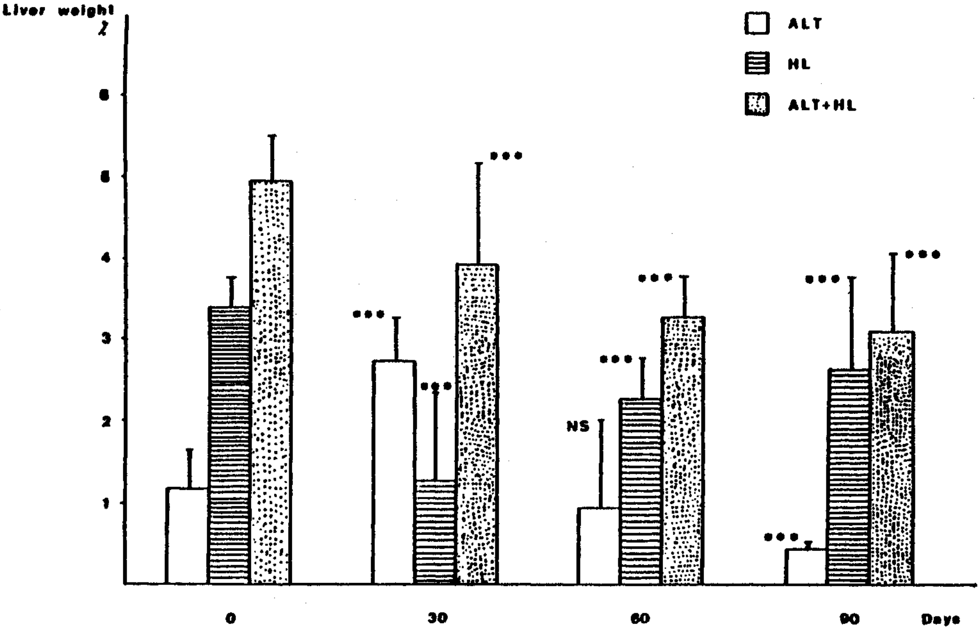

**Figure 4** Weights of the auxiliary liver transplant (ALT), host liver (HL) and total hepatic mass (ALT-HL) per cent body weight at 0, 30, 60 and 90 days at the transplantation. *** - $P < 0.001$ value statistical significant respect corresponding preoperative.

### *Histopathology*

### *Group A*

Lobular structure of graft livers in this group was preserved. There was only a slight cellular infiltration by plasma cells and lymphocytes of the portal tracts. The small arterioles were hyalinized. Moreover, many portal tracts presented a moderate degree of bile duct proliferation (Figure 5). The host livers showed uniform parenchymal atrophy and portal tracts edema.

### *Group B*

The livers grafted showed normal lobular structure, however the portal tracts were long and presented an intense degree of bile duct proliferation and a moderate cellular inflammatory infiltration (Figure 5). The host livers showed uniform parenchymal atrophy, and portal tracts edema.

### *Group C*

In all cases, the auxiliary liver grafts showed a loss of lobular structure with a severe degree of bile duct proliferation and moderate cellular infiltration of the portal tracts (Figure 5). The host livers showed parenchymal atrophy and portal tract infiltration by plasma cells and lymphocytes.

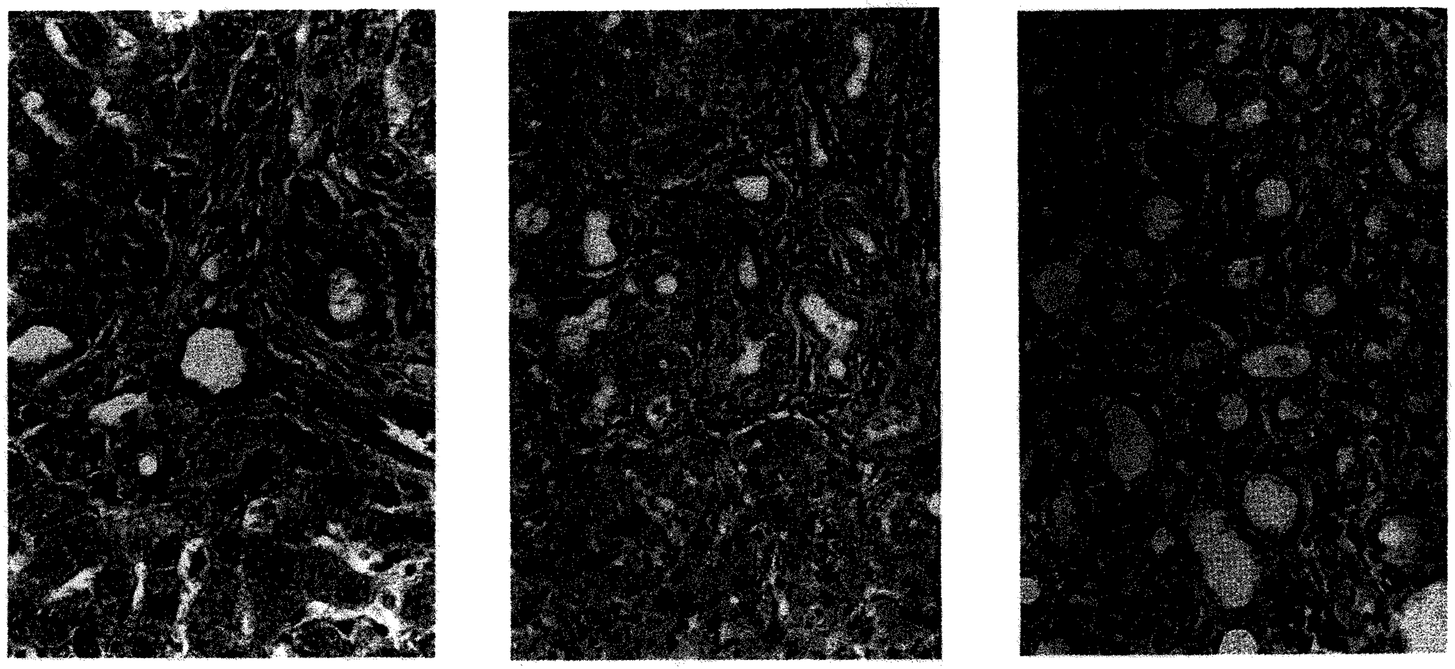

A B C

**Figure 5** Microphotographs of isografts in groups A (a), B (b) and C (c). there is a mild ductular proliferation in group A (HE × 400), intense ductular proliferation in group B (HE × 200) and severe ductular proliferation in group C. (HE ×200)

### DISCUSSION

The surgical technique used in this work was chosen after having tried other methods. On using the suprahepatic-IVC venous drainage of the graft, the results of Kort *et al.*,[10] were corroborated since all the animals died from graft congestion and had a high post-operative mortality. However, the venous drainage by the infrahepatic-IVC prevented the graft congestion and increased the post-operative survival rate. There were no pathological alterations in the central vein or sinusoids implying out-flow block in any animal in neither immediate nor long term p.o. No changes in the graft volume, consistency or color, typical of this problem, could be found in any case, but were evident in the preliminary series where the ALT drain was carried out by the suprahepatic-IVC.

For the end-to-end portal anastomosis, we used the cuff technique described by Marni and Ferrero (1985)[12] because of its simplicity, the fact that it is easy to learn and that it reduces the portal vein clamping time. These authors also used the cuff technique for the donor infrahepatic-IVC in an end-to-end fashion with the right renal vein, but that involves a right nephrectomy.

Another method used to insert the graft using the cuff technique is that proposed by Inoue *et al.* (1985)[11] who surrounded the hole of the recipient IVC by a purse-string suture through which the donor infrahepatic-IVC cuff was introduced. However, this technique requires also a right nephrectomy, and has not proved successful for us.

The donor hepatectomies (70%) reduce the graft mass, facilitate the recipient operation and diminish the complications secondary to the graft's revascularization.

There is an increase in the isograft weight during the first 30 days in a similar percentage to those described by other authors[11]. Latter, there is atrophy which is preceded by collateral circulation.

The pattern and time course of the development of collaterals were different from those described by Canty *et al.*[16] in a model of extra-hepatic portal hypertension in rats, consisting in lymphatic ligation, and gradual portal venous occlusion with an aneroid constrictor. In this model, the lumen of the portal vein is totally occluded by two weeks. This difference could probably be explained by the fact that the portal hypertension in the graft would be more slowly and gradually established.

The degree of collateral circulation in ALT is correlated with the graft atrophy and the biliary proliferation, this is greater at 90 days after operation with a subtotal replacement of the hepatic parenchyma. A similar evolution was described by Lee and Edgington[9] in syngenic partial liver transplants in rats with only hepatic artery afferent circulation and common bile-duct ligation.

Kamada and Calne[14] described a technique of orthotopic liver transplant in the rat, in which the graft only received portal flow; however, he did not attribute any pathology to the arterial hepatic ischemia. On the contrary, other authors[17-18] recommended the hepatic artery anastomosis, because a stenosis or bile duct occlusion occurred with bile duct proliferation in their long term studies.

Most experiments in rats do not include portal and arterial vascularization because of the problem of realizing both anastomosis, however Engemann established its significance in the orthotopic liver transplantation in rats.

The tolerance of absent arterial flow in the rat could be due to the different distribution of hepatic arterial vascularization, in different species[19].

The cholecho-duodenostomy is the origin of many problems[11-17] such as biliary leakage, peritonitis, central necrosis of the liver and local infection. Moreover, choledocho-duodenostomy has been used by other authors[12-20] with excellent results. The use of choledocho-gastrostomy which is theoretically more sterile does not seem to avoid a slight biliary proliferation at 30 days of tansplantation[11].

Although the graft is revascularizated with portal flow, the collateral circulation due to portal hypertension causes the atrophy of the graft since the hepatotrophic factors are diverted to the systemic circulation.

Those diverted away from the graft through the portosystemic collateral circulation might reach the host liver through the hepatic artery and thus prevent its atrophy, but this circulation provokes graft atrophy when the regenerating stimulus ends.

This hypothesis is supported by the results of Rozga *et al.*[7-8] which show that in rats with left portal branch ligation an porto-systemic shunt, the portal borne hepatotrophic factors undergo a systemic recirculation and affect the liver through the hepatic artery, decreasing the atrophy in the ligated liver lobes by systemic recirculation of the hepatotrophic portal factors.

Therefore, the auxiliary partial liver transplantations in the rat with only portal flow can only be considered as a temporary support of the hepatic function, since the portal hypertension and porto-systemic collaterals determine a systemic redistribution of the hepatotrophic portal factors.

### *References*

1. Fortner, J.G., Kim, D.K., Shiu, M.H., Yeh, S.D.J., Howland, W.S., Beattie, E.J. (1977) Heterotopic (auxiliary) liver transplantation in man. *Transplant. Proc.* **9**, 217–221
2. Houssin, D., Franco, D., Berthelot, P., Bismuth, H. (1980) Heterotopic liver in end-stage HBsAg-positive cirrhosis. *Lancet*, **1**, 990–993
3. Chandler, J.G., Lee, S., Krubel, R., Rosen, H., Nakaji, N.T., Orloff, M.J. (1971) The inter-liver competition and portal blood in regeneration of auxiliary liver transplants. *Sur. Forum* **22**, 341–343
4. Van der Heyde, M.N., Schalm, L., Vink, M. (1967) The role of functional competition in auxiliary liver transplantation. *Transplantation* **5**, 78–80

hepatic allograft function. *Surg. Gynecol. Obstet.* **135**, 769–776

6. Starzl, T.E., Porter, K.A., Kashiwagi, N., Putnam, C.W. (1975) Portal hepatotrophic factors, diabetes and acute liver atrophy, hypertrophy and regeneration. *Surg. Gynecol. Obstet.* **141**, 843–858
7. Rozga, J., Jeppson, B., Bengmark, S. (1986) Portal branch ligation in the rat. Reevaluation of a model. *Am. J. Pathol.* **125**, 300–308
8. Rozga, J., Jeppson, B., Bengmark, S. (1986) Hepatotrophic effect of portal blood during hepatic arterial recirculation. *Eur. Surg. Res.* **18**, 302–311
9. Lee, S., Edgington, T.S. (1968) Heterotopic liver transplantation utilizing inbred rat strains. I. Characterization of allogeneic graft rejection and the effects of biliary obstruction and portal vein circulation on liver regeneration. *Am. J. Pathol.* **52**, 649–669
10. Kort, W.J., Wolff, E.D., Eastham, W.N. (1971) Heterotopic auxiliary liver transplantation in rats. Use of the infrahepatic vena cava as the efferent vessel. *Transplantation* **12**, 415–420
11. Inoue, S., Kawano, N., Morioka, Y. (1985) Cyclosporine and partial liver allotransplants in a simplified rat model. *Jpn. J. Surg.* **15**, 299–311
12. Marni, A., Ferrero, M.E. (1985) Heterotopic liver grafting in the rat. A simplified method using cuff techniques. *Transplantation* **39**, 329–330
13. Higgins, G.M., Anderson, R.M. (1931) Experimental pathology of the liver of the white rat following partial surgical removal. *Arch. Pathol.* **12**, 186–202
14. Kamada, N., Calne, R.Y. (1983) A surgical experience with five hundred thirty liver transplants in the rat. *Surgery* **93**, 64–69

15. Lee, S., Edgington, T.S., Orloff, M.J. (1969) The role of afferent blood supply in regeneration of liver isografts in rats. *Surg. Forum* **19**, 360–362
16. Canty, T.G., Jauregizar, E., Fernandez-Cruz, L. (1980) Experimental portal hypertension in the rat. *J. Pediatr. Surg.* **15**, 819–825
17 Engeman, R. (1985) Technique for othotopic rat liver transplantation. In A. Thiede, E. Deltz, R. Engemann, H. Hamelmann (Eds). Microsurgical models in rats for transplantation research pp. 69–75. Berlin: Springer-Verlag
18. De Pedro, J.A., Brandau, D., Delgado, M.A., Aller, M.A., Jimenez, G., Rodriguez, J., Arias, J. Duran, H. (1986) A surgical experience with fifty liver transplantations in the rat. *Ann. N.Y. Acad. Sci.* **463**, 278–280
19. Yamamoto, K., Sargent, P.A., Fishei, M.M., Yousou, J.H. (1986) Periductal fibrosis and lipocytes (Fat-storing cells or Ito Cells) during biliary atresia in the Lamprey. *Hepatology* **5**, 452–456
20. Tersptra, O.T., Reuvers, C.B., Kooy, P.P.M., Ten Kate, F.J., Jeekel, J. (1983) Auxiliary transplantation of a partial liver graft in the dog and in the pig. *Neth. J. Surg.* **35**, 188–191

(*Accepted by S. Bengmark on 1 December 1989*)

### INVITED COMMENTARY

Heterotopic auxiliary liver transplantation has received renewed attention through general advances in clinical liver transplantation, although fundamental questions about the long-term functional capacity of an auxiliary heterotopically placed allograft yet need to be investigated. Two major developments are triggering the research in auxiliary liver grafting: (1) The need for a treatment of patients believed to be too sick for receiving orthotopic replacements of their livers and, (2) The availability of segmental or reduced size liver transplantation for treating pediatric and adult recipients, facilitate orthotopic or heterotopic auxiliary liver transplants.

The first problem has been reapproached by a group that has a long tradition in performing heterotopic liver grafting experimentally. Applying one fundamental fact, i.e., the requirement of protal venous blood supply for the graft, the group of Terpstra, Schalm and Van der Heyde, shared the portal venous blood between the cirrhotic liver and the normal liver graft. The high portal venous pressure – caused by the cirrhotic host liver – quasi guarantees the adequate blood flow to the transplant. In this aspect, the human setting is unique and hardly investigatable in an animal model. What is importantly derived from animal experiments is the finding that the host liver has to be at a disadvantageous situation – functionally and hemodynamically -- to allow the transplant to regenerate. Ligation of the host bile duct or occlusion or deviation of the portal vein provides such a disadvantage.

Applying another prerequisite for successful heterotopic liver grafting, i.e. adequate venous outflow, it appears that long term auxiliary grafting is indeed a practical option in the animal setting and clinical transplantation.

The paper by Lorente, *et al.* advocates the use of the subhepatic vena cava as an outflow provision – addressing this important problem – in contrast to using a suprahepatic segment of the vena cava as it has been used by Lee, *et al.* (Lit.). The long term survival of the transplants strongly support this modification in the model.

Secondly, whenever auxiliary liver grafting is performed successfully, small donor organs or segments are used as it is suggested in animal models. The main reason is the lack of space to accommodate a full sized organ. However, another reason is the fact that a reduced size graft requires less blood supply as compared to a large organ. Thereby, the initial portal blood sharing with the host liver, provides sufficient blood flow to the small graft allowing for hemodynamic accommodation, adequate perfusion and – possibly regeneration This is exactly the point where further studies are needed to determine the mechanism of initiation and regulation of hepatic regeneration in auxiliary transplants. The complex problem has already been addressed several years ago but, a rather confusing terminology has evolved, describing inter-liver competition for hepatotrophic, hepato-protective or even atrophy preventing substances.

This paper is not offering a clue but it adds two pieces of information to those who study hepatic regeneration.

1. The model presented here is quite adequate to study the initial phenomena of reduced size liver allografting in a biological setting, pointing out that the rat model is limited to short term experiments. As soon as the graft undergoes changes that leads to increase of its vascular resistence, collaterals inadvertently develop, reestablishing portal blood flow to the host liver via direct or arterial recirculation.
2. One phenomenom is described, though not explicitly mentioned, which is the consistency of the overall functioning liver mass, regardless of how many livers are placed into the individual. The total hepatic mass, in per cent of body weight, tends to not exceed the original liver mass (host liver) at the beginning of the experiment. After 30 days the total liver weight (graft plus host liver) is presumably not significantly different from the original host liver weight – which resembles the original biological balance. After 60 and 90 days, respectively, there is definitely no difference detected (Figure 4) indicating that the body balances out its required liver mass regardless of how many livers or liver segments are in the abdomen.

The work of Lorente, *et al.* present an initial set of experiments and require more data to study the inter-liver competition phenomena. However, one methodological principle should be stressed when employing liver iso- or allograft for morphological, functional and imunological studies: Advances in microsurgery in the rat employing vascular or cuff techniques for anastomoses allow for full revascularization of the grafts, including re-anastomoses of the hepatic artery. Since the detailed investigation of this model by Engemann and the later Zimmermann, it is virtually unacceptable to investigate the fate of orthotopic or auxiliary liver grafts without providing arterial blood supply. It is conceivable that proliferative changes that lead to graft fibrosis in the settings described by Lorente, *et al.* are attributed to the pitfall in their technique. Long term observations are clearly hampered by the incomplete revascularization of the transplant. Furthermore, analogies to human experience can only be drawn if the graft's vascular supply is identical to the clinical setting. From there it has been shown that the lack of arterial supply is detrimental to the graft survival.

Christoph E. Broelsch
Department of Surgery
University of Chicago
Chicago Ill. USA

### *References*

1. Lee, S., Broelsch, C.E., Flamant, Y.M., Chandler, J.G., Charters, A.C. and Orloff, M.J. (1975) "Liver regeneration after portacaval transportation in rats. *Surgery* 77(1), 144–149
2. Orloff, M.J., Lee, S., Charters, A.C., Chandler, J.G., Sgro., J.C., Grambort, D.E. and Broelsch, C.E. (1974) "Site of origin of the hepatotrophic portal blood factor responsible for liver regeneration. *Gastroenterology* (**66**), A–101/755
3. Schalm, L., Bax, H.R. and Mansens, B.J. (1956) "Atrophy of the liver after occlusion of the bile ducts or portal vein and compensatory hypertrophy of the unoccluded portion and its clinical importance. *Gastroenterology*, **31**, 131
4. Van der Heyde, M.N. and Schalm, L. (1968) "Auxiliary liver-graft without portal blood. Experimental autotransplantation of left liver lobes. *Brit. J. Surg.* 55(2), 114–118

